# Genetic Abnormalities in Biliary Brush Samples for Distinguishing Cholangiocarcinoma from Benign Strictures in Primary Sclerosing Cholangitis

**DOI:** 10.1155/2016/4381513

**Published:** 2016-04-03

**Authors:** Margriet R. Timmer, Chiu T. Lau, Sybren L. Meijer, Paul Fockens, Erik A. J. Rauws, Cyriel Y. Ponsioen, Silvia Calpe, Kausilia K. Krishnadath

**Affiliations:** ^1^Department of Gastroenterology and Hepatology, Academic Medical Center, 1100 DD Amsterdam, Netherlands; ^2^Center for Experimental and Molecular Medicine, Academic Medical Center, 1100 DD Amsterdam, Netherlands; ^3^Department of Pathology, Academic Medical Center, 1100 DD Amsterdam, Netherlands

## Abstract

*Background*. Primary sclerosing cholangitis (PSC) is a chronic inflammatory liver disease and is strongly associated with cholangiocarcinoma (CCA). The lack of efficient diagnostic methods for CCA is a major problem. Testing for genetic abnormalities may increase the diagnostic value of cytology.* Methods.* We assessed genetic abnormalities for* CDKN2A*,* TP53*,* ERBB2*,* 20q*,* MYC*, and chromosomes 7 and 17 and measures of genetic clonal diversity in brush samples from 29 PSC patients with benign biliary strictures and 12 patients with sporadic CCA or PSC-associated CCA. Diagnostic performance of cytology alone and in combination with genetic markers was evaluated by sensitivity, specificity, and area under the curve analysis.* Results*. The presence of* MYC* gain and* CDKN2A* loss as well as a higher clonal diversity was significantly associated with malignancy.* MYC* gain increased the sensitivity of cytology from 50% to 83%. However, the specificity decreased from 97% to 76%. The diagnostic accuracy of the best performing measures of clonal diversity was similar to the combination of cytology and* MYC*. Adding* CDKN2A* loss to the panel had no additional benefit.* Conclusion*. Evaluation of* MYC* abnormalities and measures of clonal diversity in brush cytology specimens may be of clinical value in distinguishing CCA from benign biliary strictures in PSC.

## 1. Introduction

Primary sclerosing cholangitis (PSC) is a cholestatic liver disease characterized by diffuse inflammation and progressive stricturing of the intra- and extrahepatic bile ducts, which may eventually lead to liver cirrhosis. Patients with PSC have an increased risk of developing cholangiocarcinoma (CCA) with a lifetime incidence of approximately 10–20%. Accurate detection of CCA is of paramount importance to select patients for curative treatment options such as surgical resection or liver transplantation [1, 2]. Therefore, patients presenting with a dominant biliary stricture should be thoroughly evaluated for the presence of a CCA [3]. The diagnostic approach includes imaging modalities, serum levels of the tumor marker Ca19-9 as well as biopsies, and brush cytology of the bile ducts obtained during endoscopic retrograde cholangiopancreatography (ERCP). However, benign inflammatory strictures that are part of the natural history of PSC are often difficult to distinguish from malignant strictures. Imaging techniques and Ca19-9 have limited sensitivity, while increased Ca19-9 levels can also be found in PSC in the absence of malignancy [4–6]. Conventional cytology is highly specific (95%–100%) for diagnosing CCA, but its sensitivity is generally low and varies between 12% and 62% [7–10]. The use of molecular markers could improve the diagnostic value of cytology.

Genetic abnormalities that are involved in the process of malignant progression provide a source of potential biomarkers [5, 11–14]. In PSC, both the release of inflammatory cytokines and the toxic effect of bile acids during cholestasis seem to contribute to the occurrence of genetic abnormalities and malignant transformation of cholangiocytes [15–17]. The diagnostic value of DNA content abnormalities in brushing specimens has been evaluated in several studies using different techniques including fluorescence* in situ* hybridization (FISH) and flow cytometry and has been shown to have a sensitivity of around 50% [5, 18]. The use of DNA content abnormalities is mainly based on the assessment of chromosome copy number alterations as a diagnostic feature of malignancy, whereas reports on the assessment of losses or gains of particular tumor suppressor genes or oncogenes are scarce [19, 20].

Measurement of clonal diversity is a novel method that can be used to risk stratify precancerous lesions. It is assumed that cancers evolve by a reiterative process of clonal expansion, genetic diversification, and clonal selection, which also depends on environmental conditions. Although cancer clonal dynamics can be complex and result in highly variable patterns of genetic diversity, innovative measures of clonal diversity have been shown to be of prognostic value in several malignant and premalignant conditions including Barrett's esophagus and esophageal adenocarcinoma [21]. As far as we know these measures have not been studied in PSC.

Because reliable markers for detection of CCA are currently not available, we aimed to evaluate a novel panel of DNA FISH markers and markers of clonal diversity in cytology specimens of patients with PSC, PSC-associated CCA, and CCA to further clarify the process of malignant degeneration and see if they could serve as tumor markers with the aim of improving clinical management. Our panel included the centromeric probes of chromosomes 7 and 17, locus-specific probes to the tumor suppressor genes* CDKN2A* (*p16*) and* TP53,* and three oncogenes including* ERBB2* (*Her-2/neu*),* 20q*, and* MYC*. In previous studies, all seven markers have been shown to be common alterations in bile duct malignancy, although the frequencies vary widely depending on the techniques used [17, 19, 22–27]. In addition, we tested if, based on this set of markers, measures of genetic clonal diversity have potential for diagnosing malignancy in biliary strictures.

## 2. Materials and Methods

### 2.1. Study Design and Patients

We prospectively included all consecutive patients with well-defined PSC with suspected biliary strictures who underwent ERCP in our tertiary referral center between January 2012 and April 2014. The diagnosis of PSC was based on the presence of characteristic bile duct changes including multifocal strictures and segmental dilatations on cholangiography (ERCP or magnetic resonance cholangiopancreatography) and a compatible cholestatic biochemical profile, after exclusion of other causes of secondary sclerosing cholangitis. In addition, brush cytology specimens were obtained from patients with sporadic CCA, while negative control specimens were obtained from the duodenum of patients who underwent upper endoscopy to evaluate upper gastrointestinal symptoms during which no endoscopic abnormalities were found. Demographic data and corresponding cytological results were recorded in a prospectively maintained database. The local ethics committee approved the study and all patients provided informed consent.

### 2.2. Specimen Collection and Conventional Cytology

**Figure 1:**
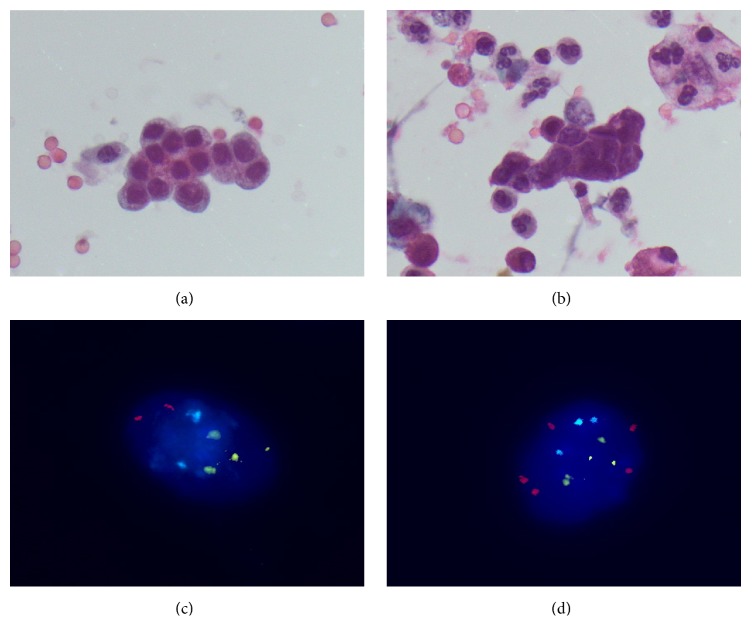
Representative examples of conventional cytology and DNA FISH of biliary brushing specimens. ((a) and (b)) Conventional cytology. (a) Reactive changes in the context of PSC with some architectural irregularity, mild variation in nuclear size, and overall intact nuclear to cytoplasmic ratio. (b) Adenocarcinoma in a patient with underlying PSC showing hyperchromatic nuclei, marked variation in nuclear shape and size with nuclear molding, and severely disturbed nuclear to cytoplasmic ratio. In the background, necrotic debris with degenerative cells and granulocytes are observed. ((c) and (d)) Representative examples of FISH signal patterns seen in biliary strictures with probes for* CEP7* [aqua],* CEP17* [green],* 20q* [gold], and* MYC* [red]. (c) A normal cell (2 signals of each probe) and (d) gain of* MYC* (>2 red signals) and gain of* CEP7* (>2 blue signals).

Cytology samples were taken from the strictures using a cytology brush compiled of stiff and soft bristles (Infinity*™*, US Endoscopy, Mentor, OH) by moving the brush several times back and forth through the stricture. The brush was cut into two equal parts and both were placed in a separate vial with 20 mL PreservCyt solution (Hologic, Marlborough, MA). The first vial was used for conventional cytology and analyzed per standard practice by a pathologist. Cytologic diagnoses were categorized as “negative,” “atypical,” “suspicious,” or “malignant” using earlier published criteria [28, 29]. Representative examples of biliary brushing specimens are shown in Figures 1(a) and 1(b).

### 2.3. Fluorescence* In Situ* Hybridization

Cells were released from the brush by rigorous shaking and concentrated in 3 mL by removal of the supernatant. The cytospin procedure (Shandon Cytospin 4, Cytocentrifuge, Thermoscientific, Waltham, MA) was performed to concentrate the cells on a slide in a uniform monolayer after which the slides were stored at −80°C until further preparation for FISH. FISH was performed as previously described using seven different DNA probes including the centromeric probes* CEP7* and* CEP17* and the locus-specific probes to* CDKN2A*,* TP53*,* ERBB2*,* 20q*, and* MYC* (Abbott Molecular, Abbott Park, Illinois) [30]. Probes were organized in two probe sets with set 1 comprising* CEP17*,* ERBB2*,* TP53*, and* CDKN2A* and set 2 comprising* CEP7*,* CEP17*,* 20q*, and* MYC*. Slides were reviewed by an experienced technician who was blinded to the patient's clinical history and cytology results. Slides were analyzed by manual counting of the number of signals for each of the probes in 100 (minimum 75) consecutive interphase nuclei of biliary epithelial cells. Nuclei with two signals for a particular probe were considered as normal. Data were recorded as the percentage of cells showing genetic abnormalities for a particular marker. For control purposes (to determine background hybridization variation), the probes in this study were also applied to duodenum brushes of 10 healthy control individuals. Cut-off values were calculated for each probe separately as the mean percentage of cells in duodenum specimens showing abnormalities plus twice the standard deviation, rounded up to the next integer. All samples were scored as positive or negative for each marker based on the cut-off values for each individual probe. Cut-off values were 1% (*CDKN2A* loss), 3% (*TP53* loss), 0% (*ERBB2*), 1% (*20q*), 3% (*MYC*), 3% (*CEP7*), and 4% (*CEP17*). Representative examples of DNA FISH of biliary brushing specimens are shown in Figures 1(c) and 1(d).

### 2.4. Genetic Clonal Diversity

Measures of clonal diversity were computed to quantify the degree of genetic diversity within a sample. Clones were defined as the collection of cells characterized by the same genotype based on the combination of marker-specific copy numbers for the two probe sets used in this study. Thus, for each cell, the number of signals for each probe in a particular probe set was recorded and these data were used to identify different clones. We analyzed two indices of clonal diversity for both probe sets: richness and the Shannon index. Richness measures the number of different clones, while the Shannon index takes into account both the number of different clones and the abundance of clones [21]. The Shannon diversity index (*S*) is calculated as *S* = ∑_*i*=1_^*R*^ *p*_*i*_ln⁡(*p*_*i*_), where *R* is the number of different clones in a sample and *p*_*i*_ is the frequency of clone *i* in the sample.

### 2.5. Gold Standard for Malignancy

Pathological diagnosis was considered the “gold standard” for diagnosing benign and malignant disease. Pathological evidence of carcinoma included positive routine cytology, histological confirmation of a CCA, or positive fine needle aspiration indicating metastatic disease. Strictures were classified as benign based on negative pathology results and a cancer-free clinical course of at least six months.

### 2.6. Statistical Analysis

All statistical analyses were performed using SPSS (version 21) and GraphPad Prism (version 5.01). Data were presented as mean ± standard deviation (SD) or median and interquartile range (IQR) for quantitative variables and frequencies and percentages for categorical data. Groupwise comparisons of categorical data were analyzed by using Fisher's exact test and continuous measures were compared with the use of Student's *t*-test. Diagnostic performance of cytology alone and in combination with genetic markers was evaluated by sensitivity, specificity, and area under the curve (AUC) analysis. Receiver operator characteristic (ROC) curves were created to determine optimal cut-off values for different measures of diversity. Results were considered statistically significant at *P* values < 0.05.

## 3. Results

### 3.1. Patients

Brushes were obtained from 41 individual patients undergoing ERCP including 29 patients with PSC, 3 with PSC complicated by CCA, and 9 with CCA. In the majority of patients (97%) a dominant stricture was found during ERCP. All patients had corresponding cytology and FISH results. Patients reported complaints of jaundice (51%), fatigue (39%), abdominal pain (29%), and weight loss (24%). Most patients (81%) reported at least one of the above-mentioned complaints. Patient characteristics and laboratory values at the time of ERCP are shown in Table 1. Within the group of PSC patients (*n* = 32, 17 males, median age 47 years), malignancy was confirmed by conventional cytology (*n* = 1), detection of metastatic disease by fine needle aspiration (*n* = 1), and, in one patient with a high suspicion of a CCA, the final diagnosis made at the time of surgical resection. In the remaining 29 PSC patients with a stricture classified as “benign,” no malignancy was diagnosed during follow-up (median follow-up, 16 months, IQR, 8–27 months). The population of patients with a sporadic CCA consisted of 4 male and 5 female patients (mean age 63.4 ± 12.5 years).

### 3.2. Conventional Cytology

Overall, 26 samples (63%) were classified as negative, 8 (20%) were classified as atypical, four (10%) were classified as suspicious, and three patients (7%) had a brush that was positive for CCA. When only a “malignant” cytology result was considered positive for malignancy, this resulted in an overall sensitivity of 25% and a specificity of 100% (Table 2). The sensitivity and specificity were 50% and 97%, respectively, when all specimens classified as “suspicious” or “malignant” were considered positive for malignancy. Among the PSC patients, cytology had a sensitivity of 33% and a specificity of 100%. Of the 3 patients with a PSC-associated CCA, one had a “malignant” cytology result, one was reported as “atypical,” and one brush was negative. In the PSC patients without malignancy, cytology from one patient was reported as “suspicious for malignancy.” This patient underwent multiple ERCP during follow-up for short-term stenting and balloon dilatation of the stricture, but additional bile duct brushing (3 times) during a period of 19 months did not show any more signs of malignancy.

### 3.3. Genetic Abnormalities

First, we compared the presence of genetic abnormalities in malignant strictures (both PSC and non-PSC) to benign PSC strictures (Table 2). The markers* CDKN2A* loss and* MYC* gain were significantly associated with malignancy and were observed in 67% and 58% of the CCAs and in 31% and 21% of the benign PSC strictures (Fisher's exact test, *P* = 0.045 and *P* = 0.029, resp.). Although gains of* CEP7* and* CEP17* were more frequently observed in CCA than in benign strictures (58% versus 24% and 83% versus 55%, resp.), these differences were not statistically significant (*P* = 0.068 and *P* = 0.154). Similarly, there was no significant difference between gains of* 20q* in malignant (67%) and benign strictures (41%) (*P* = 0.18).

We evaluated the diagnostic performance of cytology and the best performing makers (i.e., the markers* CDKN2A* and* MYC* that were both significantly associated with CCA) in different combinations. The combination of cytology and FISH for* MYC* (considered positive when either cytology or FISH was positive) resulted in an increase in sensitivity from 50% to 83%, but specificity decreased from 97% to 76%. Combining cytology with FISH for both* CDKN2A* and* MYC* increased the sensitivity further to 92%, but this was at the expense of lower specificity (52%). This was also reflected by the AUC that was 0.73 for cytology alone, 0.80 for cytology combined with FISH analysis for* MYC,* and 0.72 for cytology combined with* CDKN2A* and/or* MYC*. When we combined* CDKN2A* and* MYC* without cytology, 10 out of 12 CCA patients tested positive for at least one of the markers. However, of the 29 patients with benign strictures, also 13 patients had* CDKN2A* and/or* MYC* abnormalities. Sensitivity and specificity were 83% and 55%, respectively.

**Figure 2:**
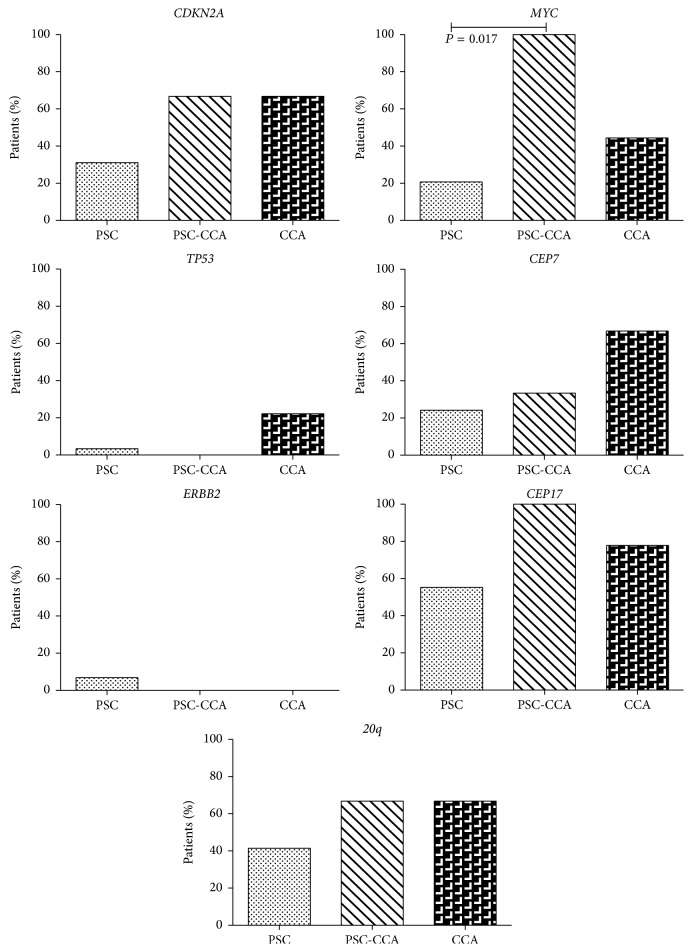
Genetic abnormalities in PSC, PSC-associated CCA, and sporadic CCA. *P* values are compared using Fisher's exact test.

When we restricted the analysis to PSC patients only, we observed that abnormalities of* CDKN2A*,* 20q*,* CEP17*, and* MYC* were more frequent in PSC-CCA than in CCA. However, only the difference in MYC abnormalities was a significant finding where* MYC* abnormalities were seen in 21% of benign PSC cases and in all cases of PSC-CCA (*P* = 0.017) (Figure 2). This corresponded to a sensitivity of 100% and a specificity of 79%. Interestingly,* MYC* abnormalities were also seen more frequently in PSC-CCA than in sporadic CCA (100% versus 44%). This difference was, however, not significant (*P* = 0.21).

### 3.4. Clonal Diversity

**Figure 3:**
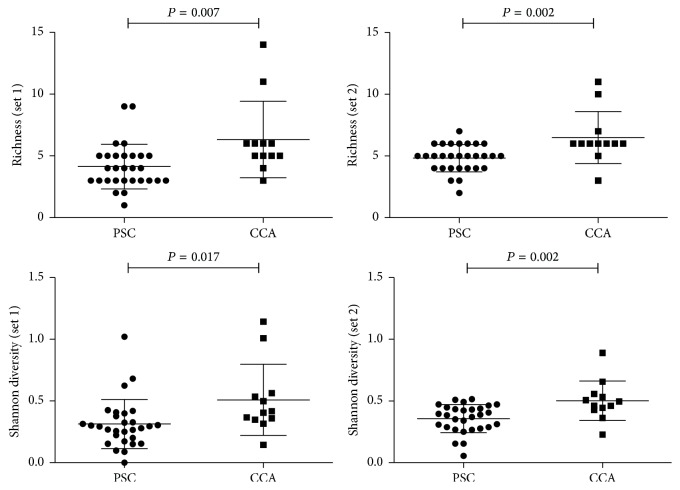
Genetic clonal diversity in primary sclerosing cholangitis and cholangiocarcinoma. *P* values are compared with use of Student's *t*-test.

The number of different clones per sample (i.e., richness) was significantly higher in CCA compared with PSC. This was the case for both probe set 1 (mean 6.33 versus 4.14, *P* = 0.007) and probe set 2 (mean 6.50 versus 4.83, *P* = 0.002) (Figure 3). Also the Shannon diversity was significantly higher in CCA than in PSC when measured by probe set 1 (mean Shannon diversity of 0.51 versus 0.31, *P* = 0.017) and probe set 2 (mean 0.50 versus 0.36, *P* = 0.002). We computed ROC curves to compare the discriminative performance of the different measures of diversity. Richness (set 2) and Shannon diversity (set 1) had the highest discriminative value with both having an AUC of 0.78 (Table 2). Using a cut-off value of 5.5 for richness (set 2) and a cut-off value of 0.34 for Shannon diversity (set 1), this corresponded to a sensitivity of 83% and a specificity of 72%. Both measures increased the sensitivity of normal cytology from 50% to 92%, although specificity went down to 69%. Cytology and richness (set 2) combined had an AUC of 0.80 and cytology combined with Shannon diversity (set 1) also resulted in an AUC of 0.80. However, the best performing diversity measures already had an AUC of 0.78 by itself.

## 4. Discussion

Brush cytology has limited sensitivity for diagnosing CCA as the cause of biliary strictures, which is important because accurate diagnosis of early-stage CCA could increase the number of patients suitable for surgical resection or liver transplantation. Surveillance is therefore predicated on the availability of reliable diagnostic tools. Because most CCAs develop in the perihilar region or in the extrahepatic bile ducts, brushing and biopsy specimens of the biliary tract still represent the most reliable method for early detection of malignant degeneration. CCAs are often surrounded by dense, desmoplastic stroma and in a significant number of cases cytology specimens do not contain sufficient epithelial cells for interpretation. The use of FISH enables the detection of genetic abnormalities, even when these abnormalities are only present in a small fraction of cells, and FISH is therefore an attractive diagnostic tool.

Previous studies have revealed several genetic abnormalities involved in malignant progression in CCA. However, studies often focused on single genetic events, while the assessment of genetic diversity may provide additional information about the genetic profile of the disease, which cannot be obtained from a single marker. This may ultimately lead to improved diagnostic and prognostic tools. Multicolor DNA FISH on cytology specimens as applied in this study is an excellent method for identifying subclones in cellular specimens, which can be used to determine diversity status. Therefore, we not only assessed conventional biomarker status of single events but also used the frequency and abundance of genetic alterations at multiple loci to measure indices of clonal diversity.

We included 29 patients with well-defined PSC and 12 patients with CCA. Patients were prospectively followed up during a median of 16 months to assure that all patients without CCA were truly “benign” patients. We observed that abnormalities of* MYC* and* CDKN2A* were significantly associated with the presence of malignancy. As expected, abnormalities of* CEP7*,* CEP17*, and* 20q* were more frequently noticed in malignancy, but the differences between the benign and malignant cases were not statistically significant in our dataset. Combining cytology with* MYC* aberrations increased the sensitivity from 50% to 85%, however with a decrease in specificity from 97% to 76%. As has been previously reported,* CDKN2A* loss was also significantly associated with CCA but it did not improve the diagnostic performance beyond the use of* MYC* [19].

Furthermore, we demonstrated for the first time that levels of clonal diversity are significantly higher in CCA compared to benign PSC strictures. This is in line with previous studies on premalignant diseases such as Barrett's esophagus where an increase in genetic diversity is observed as patients approach the diagnosis of esophageal adenocarcinoma [21, 31]. Interestingly, these findings were independent of the probe set used for the diversity analysis. The best performing measures of clonal diversity were richness (set 2) and the Shannon index (set 1). They had an AUC that was similar to the diagnostic accuracy of the combination of cytology and* MYC*. The use of cytology combined with diversity measures did not yield substantial improvement and the AUC only slightly increased from 0.78 to 0.80 for both measures.

The diagnostic value of* MYC* abnormalities is in accordance with the findings of a recent study that evaluated a novel pancreatobiliary FISH probe set in a large cohort and compared it to the UroVysion probe set [32]. The probes in the panel (1q21, 7p12, 8q24, and 9p21) were first evaluated on 30 selected samples of tumor tissue (CCA and pancreatic cancer) where gain of 8q24 (*MYC*) was observed in 44% of the tumor cells analyzed. Subsequently, the authors performed a retrospective analysis of brush samples from 272 patients who underwent ERCP for evaluation of malignancy and found that the presence of polysomy (≥5 cells in a sample with copy number gain of at least 2 of the 4 probes), assayed by the new probe set, resulted in an improved sensitivity of 65% when compared to the UroVysion probe set (sensitivity 46%) for the detection of pancreatobiliary malignancies. Because the 8q24 probe was only evaluated in combination with the other probes to diagnose polysomy, the diagnostic value of 8q24 as a single marker remained unclear.

In our study, although the number of cases was small,* MYC* had a sensitivity of 100% and a specificity of 79% for the diagnosis of CCA in PSC. The* MYC* (or c-myc) protooncogene is a regulator gene that encodes for a transcription factor and plays a central role in regulating cell cycle proliferation, neoplastic transformation, and apoptosis and* MYC* abnormalities have been found in many cancers including CCA [33]. In a mouse model of chronic cholestasis, developed to study the mechanisms by which cholestasis contributes to biliary carcinogenesis,* MYC* was upregulated during CCA progression and resulted in induction of cyclin D1, a protein that contributes to dedifferentiation and cell proliferation in CCA [34]. In addition, knockdown of* MYC* reduced progression to CCA.* MYC* abnormalities have also been described in an inflammatory setting. Komori et al. showed that stimulation by the proinflammatory cytokine tumor necrosis factor-alpha was associated with aberrant expression of the mutagenic enzyme activation-induced cytidine deaminase (AID) in human cholangiocarcinoma-derived cells resulting in somatic mutations of several genes including* TP53*,* KRAS,* and* MYC* [17]. Interestingly,* MYC* abnormalities were not only more present in malignant (PSC) strictures compared to benign strictures, but also more frequent in PSC-CCA than in sporadic CCA (100% versus 44%). Although this difference was not statistically significant (most likely due to small numbers), this may indicate that the genetic profile of CCAs arising in PSC differs from sporadic CCA due to the inflammatory setting that may play a more important role in the PSC-associated CCAs.

Our findings of* CEP17* abnormalities being associated with CCA are in line with previous studies that evaluated copy number alterations of chromosomes 3, 7, and 17 to evaluate the presence of polysomy which yields a sensitivity and specificity of approximately 51% and 93%, respectively. In contrast to our findings, previous studies have reported abnormalities of* TP53* as frequent events in CCA [35]. In our analysis, losses of* TP53* were not seen at high levels and were not significantly different between PSC and PSC-associated CCA. Part of the discrepancy may be due to differences in detection techniques. Abnormalities of* TP53* are usually point mutations, which cannot be detected by FISH, and our study may have underestimated* TP53* abnormalities. In addition, most of these studies were performed on resection specimens and may represent more advanced disease.

A major limitation of our study should be considered. Only twelve patients in our study had a confirmed CCA of which three had underlying PSC. Nevertheless, we found significant differences in genetic abnormalities between patients with malignant and benign strictures in PSC. The results of our study suggest a potential role for evaluating genetic markers on brushing specimens, which should include the evaluation of* MYC* abnormalities and measures of clonal diversity, and further studies with larger cohorts are needed to confirm our findings.

## Figures and Tables

**Table 1:** Clinical characteristics and laboratory values of patient populations studied.

	PSC	CCA
	*n* = 29	*n* = 12
Male sex	14 (48%)	7 (58%)
Age, years	43.5 ± 13.0	60.6 ± 12.0
Jaundice	11 (38%)	10 (83)
Weight loss	3 (10%)	7 (58%)
Abdominal pain	10 (35%)	2 (17%)
Fatigue	11 (38%)	5 (42%)
IBD	16 (55%)	2 (17%)
AST, U/L	86.9 ± 69.7	73.3 ± 30.9
ALT, U/L	102.0 ± 88.4	224.6 ± 196.7
ALP, U/L	365.7 ± 215.5	475.5 ± 322.8
Bilirubin *μ*mol/L	44.5 ± 57.5	157.3 ± 184.0

Values are presented as number (%) or mean ± standard deviation.

ALP: alkaline phosphatase; ALT: alanine aminotransferase; AST: aspartate aminotransferase; CCA: cholangiocarcinoma; IBD: inflammatory bowel disease; PSC: primary sclerosing cholangitis.

**Table 2:** Diagnostic performance of cytology, FISH, and measures of clonal diversity in distinguishing benign from malignant strictures.

	PSC	CCA	*P* value	Sensitivity	Specificity	AUC (95% CI)
	*n* = 29	*n* = 12				
Cytology						
Cytology (M), *n* (%)	0 (0)	3 (25)	0.021	25%	100%	0.63 (0.43–0.83)
Cytology (S + M), *n* (%)	1 (3)	6 (50)	0.001	50%	97%	0.73 (0.54–0.93)
FISH markers						
*CDKN2A* loss, *n* (%)	9 (31)	8 (67)	0.045	67%	69%	0.68 (0.49–0.86)
*TP53* loss, *n* (%)	1 (3)	2 (17)	0.20	17%	97%	0.57 (0.36–0.77)
*ERBB2* gain, *n* (%)	2 (7)	0 (0)	1.00	0%	93%	0.47 (0.28–0.66)
*20q* gain, *n* (%)	12 (41)	8 (67)	0.181	67%	59%	0.63 (0.44–0.82)
*MYC* gain, *n* (%)	6 (21)	7 (58)	0.029	58%	79%	0.69 (0.50–0.88)
*CEP7* gain, *n* (%)	7 (24)	7 (58)	0.068	58%	76%	0.67 (0.48–0.86)
*CEP17* gain, *n* (%)	16 (55)	10 (83)	0.154	83%	45%	0.64 (0.46–0.82)
Cytology and FISH						
Cytology (S + M) and *CDKN2A*, *n* (%)	10 (34)	10 (83)	0.006	83%	66%	0.74 (0.58–0.91)
Cytology (S + M) and *MYC*, *n* (%)	7 (24)	10 (83)	0.001	83%	76%	0.80 (0.64–0.95)
Cytology (S + M) and *MYC*/*CDKN2A*, *n* (%)	14 (48)	11 (92)	0.013	92%	52%	0.72 (0.56–0.88)
Diversity measures						
Richness (set 1)	11	10	0.015	83%	62%	0.73 (0.56–0.89)
Richness (set 2)	8	10	0.002	83%	72%	0.78 (0.62–0.94)
Shannon diversity (set 1)	8	10	0.002	83%	72%	0.78 (0.62–0.94)
Shannon diversity (set 2)	10	10	0.006	83%	65%	0.74 (0.58–0.91)
Diversity measures and cytology						
Cytology and richness (set 2)	9	11	<0.001	92%	69%	0.80 (0.66–0.95)
Cytology and Shannon diversity (set 1)	9	11	<0.001	92%	69%	0.80 (0.66–0.95)

*P* values are compared using Fisher's exact test. M refers to malignant and S refers to suspicious. CCA includes 3 patients with PSC-associated CCA and 9 patients with sporadic CCA. AUC: area under the curve; CCA: cholangiocarcinoma; CI: confidence interval; PSC: primary sclerosing cholangitis; NA: not applicable.

Cut-off values for measures of clonal diversity were determined using ROC curves and were 4.5 for richness (set 1), 5.5 for richness (set 2), 0.34 for Shannon diversity (set 1), and 0.43 for Shannon diversity (set 2).

## Abbreviations

CCA: Cholangiocarcinoma
PSC: Primary sclerosing cholangitis
FISH: Fluorescence* in situ* hybridization
AUC: Area under the curve
ERCP: Endoscopic retrograde cholangiopancreatography
IQR: Interquartile range
CI: Confidence interval.
